# Polio immunization in Pakistan: ethical issues and challenges

**DOI:** 10.1186/s40985-017-0049-4

**Published:** 2017-02-06

**Authors:** Sarah Basharat, Babar Tasneem Shaikh

**Affiliations:** Health Systems and Policy Department, Health Services Academy, Chak Shahzad, Park Road, Islamabad, 44000 Pakistan

**Keywords:** Polio, Immunization, Ethics, Health system, Pakistan

## Abstract

**Background:**

Immunization should be considered a basic human right to health and well-being. It is everybody’s business, and it is everybody’s responsibility: the individual, the community, the health system and the state. This paper attempts to review some of the literature that highlights the ethical and religious concerns surrounding polio vaccination and what approaches may be used to counter the problems faced in Pakistan.

**Methods:**

This paper is developed through a literature review on public health and polio in Pakistan, consulting local, regional and globally published peer reviewed articles focussing on religion, culture, ethics and public health.

**Discussion:**

Human behaviour, including the utilization and acceptability of healthcare services, is greatly influenced by religious beliefs and dogmas. Immunization, specifically for the purpose of polio eradication, has been a topic under focus and in the news in Pakistan. The government is doing its best through a variety of interventions to increase access, inform the public and increase vaccination rates. Nevertheless, the country still faces a huge challenge from certain stern pockets of uncompromising populations who resist and refuse vaccination. Beliefs, practices and cultural norms overshadow public health priorities and ethics. Understanding of the context, therefore, is critical to determine the social hindrances in polio eradication and strategize thereon.

**Conclusion:**

Having programmatic, system-wide, socio-cultural and of course ethical dimensions, the policy makers and the programme managers in Pakistan must attempt to address the multitude of challenges to polio vaccination, whereby the plan of action developed within the ethical norms could potentially lead to an ultimate success.

## Background

Immunization is considered to be the most cost-effective public health intervention having had the greatest positive impact on morbidity and mortality reduction among children under five [1]. It should be considered as one element of the basic human right to health, and hence, it is equally the responsibility of individuals, communities and the government [2]. The effectiveness of vaccination in reducing the burden of diseases has been demonstrated through research, yet the barriers to achieving 100% immunization coverage pose some serious questions and call for developing a deeper understanding of the issue [3].

Immunization greatly reduces diseases, disability and deaths [4]. Polio is a paralytic disease, which is a target for eradication, but doing so has proven to be a global challenge [5]. For those who do not receive the vaccination, due to any reason, ‘herd immunity’ developed through sufficient immunization coverage of the population helps to protect them [6]. Nonetheless, it has been documented that the utilization of healthcare services and the attitudes of a community towards immunization are greatly influenced by religious beliefs [7]. Religion and religiosity act as guidelines for many practices around the world, which include health-related practices and life choices that influence health [8]. Healthcare workers and researchers are continuously attempting to incorporate the element of cultural and religious sensitivity in provision of services and delivery of interventions in an attempt to make these services and interventions more acceptable [9].

This paper is based on a critical review of the literature with point of interest on the ethical and religious issues around polio vaccination in the local context i.e. Pakistan, and endeavours to put forth suggestions to address the same.

## Methodology

A literature search was conducted over the internet, accessing the most relevant articles on public health and polio in Pakistan, articles with focus on religion, culture and public health, articles discussing ways and means to eradicate polio from the world, and ethics and public health. We consulted Google Scholar for the MeSH terms [Polio, Ethics, Health systems, Public health, Pakistan], and the full texts of articles of our choice were retrieved from MEDLINE/PubMed, where available.

## Discussion

In recent times, conflict and insecurity have been particularly damaging to polio eradication efforts. Governments and NGOs have been reluctant to provide humanitarian assistance to refugees and internally displaced persons unless they agreed to polio immunization for their children; this has eventually raised questions and ethical challenges [10]. Does the state have the right to impose such vaccinations in instances where the serious vaccine-preventable disease could threaten local natives, health workers and other personnel? There are ‘justificatory conditions’ to help establish when the moral considerations vital to public health (preventing and removing harms, benefiting others and utility) can take precedence over other goals, if such a need arises during certain public health interventions and activities. These conditions have been termed as proportionality of the activity (probable public health benefits outweigh other moral considerations), necessity of the activity, effectiveness of the activity, the extent to which the activity represents the least infringement of other moral considerations and the ability to publicly and openly justify the activity [11]. Since immunization is a public health programme, it has been suggested that it falls within this ethical framework. Moreover, as vaccination has a direct positive impact on the health of the community and not just the person being vaccinated; unvaccinated people could therefore be said to be causing a direct ‘harm’ to the society [12].

Low literacy rates; rural, remote and hard to reach pockets of populations; religious dogmas; and misconceptions about the polio vaccine such as ‘it causes sterility in children’, ‘it contains pig fat’ and ‘vaccines used in programmes are sub-standard’ have seldom been addressed systematically [11, 13]. Another problem has been the lack of a system-wide governmental plan to eradicate polio with focus only on vaccinating children multiple times a year. This may have serious implications particularly with regard to the public’s trust in polio vaccine. This necessitates just and equitable resource allocations to local health systems, with comprehensive understanding of the values and health needs within a community [14]. Furthermore, poor management and health communication strategies have been identified as a major impediment, where provincial and district health authorities in charge of vaccination resources were not on board, nor were charged to engage with the religious and community leaders to advocate for the campaign [15]. In pursuit of achieving the global health outcome under the global polio eradication initiative, local traditional, social and cultural norms and ethics must not be undermined and undervalued.

## The Pakistani perspective

Pakistan’s health system’s challenges are compounded by a difficult geography, from the Himalayan glaciers of the north to the rough and tough terrain of Balochistan in the south, contributing to poor public sector health delivery [16]. Immunization specifically for the purpose of polio eradication has been a hot topic in the news in Pakistan lately. Pakistan is one of only three remaining countries in the world where polio still exists and most of the resistance in Pakistan against the immunization programme stems from the northern province of Khyber Pakhtunkhwa and parts of Balochistan [17]. In 2014 when India was declared polio free [18], a total of 306 cases of polio were diagnosed and recorded in Pakistan, which accounted for nearly 80% of all polio cases worldwide. In 2015, around 53 new cases were reported [19]. Out of the total cases diagnosed in Pakistan, nearly 96% were reported from the province of Khyber Pakhtunkhwa.

Global polio eradication efforts are very much dependent on Pakistan’s capacity to address the wide range of obstacles to immunization, including religious, political and socioeconomic barriers, inconsistencies in vaccine coverage, a weak health infrastructure and conflict in polio-endemic regions of the country [20]. Moreover, studies outside of Pakistan have demonstrated the strong influence of socio-cultural and especially religious factors on the decision by parents to immunize their children, and the opposition thus faced is a big challenge for healthcare providers. Divine providence and deliberate introduction of ‘disease’ inside the God created human body are some of the documented religious objections against vaccinations internationally [2]. In Pakistan too, the situation is somewhat comparable in certain known pockets of the population suffering from continuing transmission of polio. The resistance to polio vaccination is multifaceted and multifactorial and has put nearly 250,000 children living in the province of Khyber Pakhtunkhwa at risk of contracting polio, owing to a variety of reasons including refusals, inaccessibility of certain geographic areas, security concerns, lack of female vaccinators, etc. [21].

Currently, there are several key activities in Pakistan that are being carried out under the aegis of the National Programme for Immunization to increase access, inform the public and increase vaccination rates. These range from engagement with religious scholars and clergy [20], improving the cold chain, establishing a vaccine logistics management information system [22], strengthening monitoring of field activities, instituting research for evidence-based decision making, launching public campaigns focusing on behaviour change and most notably widening the outreach and coverage through female health workers, who now are accompanied by security personnel because of conflict in certain border areas of Pakistan [23, 24].

It has also been found that lack of education and information about the vaccine has contributed to negative attitudes and the stubborn stance against the vaccination [25, 26]. The Programme needs to reflect local value systems whilst en route to polio eradication. Recognition of the religious and cultural values as well as an understanding of the international political situation would be meaningful [27].

So the question is worthwhile to ponder: Is it ethical to administer a vaccine amidst people’s low level of knowledge about its benefits, and thereon their unwillingness, or is it unethical for people to refuse to protect their children against a potentially disabling disease? The issue of ethical concerns surrounding the subject of immunization is not a new one [10].

Denying a child the vaccine and as a result putting him/her at risk of falling prey to an otherwise avoidable disease that could leave him/her disabled for life can in no way be considered to be acceptable ethically. Immunization against Polio is a problem not limited to Pakistan. Active resistance to healthcare stemming from religious beliefs is a documented issue [28]. Strategies and modalities have to take this scenario into consideration. For instance, social mobilization is meant to empower and enable the beneficiaries of a programme to take charge of it [29]. Pragmatic social mobilization would be to involve community leaders and groups. This approach, however, might not be effective in areas where there is an active resistance to healthcare in the community, which is a much more intractable problem as it usually stems from cultural and religious beliefs and faith. Local grass root level organizations are another route to reach communities and create ownership. This approach has been found to be effective for issues such as water and sanitation, and childhood diseases [30], but its effectiveness for polio immunization has not yet been established and might be questionable in areas where there is a high degree of resistance against the immunization programme. Simultaneously, a hybrid approach might work, whereby goals and strategies are negotiated and formulated involving local organizations and community notables at all stages [31]. The situation in Pakistan can be assessed in the same way. Religious doctrine and cultural beliefs and attitudes take precedence over scientific evidence. In 2016, Pakistan was ranked 132 on the Global Prosperity Index out of 142 countries. Pakistan with a status of low human development ranks 147 on the Human Development Index and this too has been consistent over the last 5 years [32]. Whilst the importance of immunization against polio as part of a global campaign for polio eradication is understandably a hard concept for the masses to grasp, it is alarming as a strong contributor to the low human development index in the country.

A multi-sectoral approach involving education ministries is crucial. Maternal education is an established factor in ensuring positive health outcomes among children, and integrating immunization knowledge with maternal and child health services is absolutely critical [33]. Studies have demonstrated that women can play a vital role in shaping health seeking behaviours of the family, because of being the primary care giver and being relatively more concerned about children’s health [34]. The force of female vaccinators is inclined to communicate only with the women of the households about the importance of vaccination. However, in a patriarchal society like Pakistan, where the man is the head of the household, convincing the women alone may be less effective in reducing resistance to polio immunization. Vaccination rates tend to remain low where women are not empowered to take decisions regarding their children’s health [35]. Hence, the male heads of households will also need to be taken on board.

Moreover, the perceptions that the polio vaccine is a foreign plot with some mala fide intentions to harm the health of Pakistan’s children ought to be dealt with through an aggressive mass educational campaign. One way to do that could be to engage famous celebrities and well-known public figures to endorse the vaccination [36]. Winning back the public trust through a robust behaviour change strategy, addressing the important roots of immunization hesitancy and effectively engaging with emotions would be a way to improve immunization rates.

## Conclusions

Polio eradication in Pakistan faces peculiar challenges, having programmatic, system-wide, socio-cultural and ethical dimensions. The more policy makers and programme managers attempt to address the multitude of challenges, the more its nature as a form of social activism becomes compatible with ethical norms and could lead to ultimate success and eradication of the disease. In light of the complex setting encompassing polio vaccination, campaigning and eradication efforts in Pakistan, it is an opportune time to think of an indigenously developed mix of strategies. These plans of action must comply with ethical principles and dimensions. They must be culturally appropriate and feasible to engage with populations. Ensuring confidentiality, integrity and autonomy, a truly ethical system-wide approach could lead to voluntary participation of communities in polio vaccination campaigns.
